# α-MSH Stimulates Glucose Uptake in Mouse Muscle and Phosphorylates Rab-GTPase-Activating Protein TBC1D1 Independently of AMPK

**DOI:** 10.1371/journal.pone.0157027

**Published:** 2016-07-28

**Authors:** Cathrine Laustrup Møller, Rasmus Kjøbsted, Pablo J. Enriori, Thomas Elbenhardt Jensen, Cecilia Garcia-Rudaz, Sara A. Litwak, Kirsten Raun, Jørgen Wojtaszewski, Birgitte Schjellerup Wulff, Michael A. Cowley

**Affiliations:** 1 Steno Diabetes Center, Translational Pathophysiology, 2820 Gentofte, Denmark; 2 Section of Molecular Physiology, August Krogh Centre, Department of Nutrition, Exercise and Sports, Faculty of Science, University of Copenhagen, 2200 Copenhagen, Denmark; 3 Monash Obesity & Diabetes Institute, Metabolic Neurophysiology Laboratory, Monash University, 3168 Clayton, Australia; 4 Department of Pediatrics, Centenary Hospital for Women, Youth and Children and Australian National University, 2605 Canberra, Australia; 5 Incretin and Obesity Biology, Novo Nordisk A/S, 2760 Maaloev, Denmark; Tohoku University, JAPAN

## Abstract

The melanocortin system includes five G-protein coupled receptors (family A) defined as MC1R-MC5R, which are stimulated by endogenous agonists derived from proopiomelanocortin (POMC). The melanocortin system has been intensely studied for its central actions in body weight and energy expenditure regulation, which are mainly mediated by MC4R. The pituitary gland is the source of various POMC-derived hormones released to the circulation, which raises the possibility that there may be actions of the melanocortins on peripheral energy homeostasis. In this study, we examined the molecular signaling pathway involved in α-MSH-stimulated glucose uptake in differentiated L6 myotubes and mouse muscle explants. In order to examine the involvement of AMPK, we investigate α-MSH stimulation in both wild type and AMPK deficient mice. We found that α-MSH significantly induces phosphorylation of TBC1 domain (TBC1D) family member 1 (S237 and T596), which is independent of upstream PKA and AMPK. We find no evidence to support that α-MSH-stimulated glucose uptake involves TBC1D4 phosphorylation (T642 and S704) or GLUT4 translocation.

## Introduction

Over the past decades it has become clear that the central melanocortin system, driven by neurons expressing proopiomelanocortin (POMC) and agouti related protein (AgRP), is broadly involved in the regulation of mammalian energy homeostasis. POMC undergoes posttranslational processing to melanocyte stimulating hormone (α-, β- and γ-MSH) and adrenocorticotropic hormone (ACTH), which are melanocortin receptor (MCR) agonists with varying affinities [1, 2]. The central metabolic effects of the melanocortin system are mediated mainly by melanocortin receptor 4 (MC4R). Humans with MC4R mutations develop morbid obesity and account for 1-6% of morbidly obese individuals across diverse ethnic groups [3–7]. The obese phenotype in humans with MC4R deficiency is characterized by increased fat and lean mass [8] and early onset hyperphagia [9]. Likewise, MC4R knockout mice have increased lean body and fat mass and hyperphagia [10].

The peripheral effects of melanocortin peptides have not been as intensely studied as the central effects, even though MCRs are expressed outside of the central nervous system (CNS) [2]. The pituitary gland is the source of various POMC-derived hormones such as ACTH, β-lipotrophin, β-endorphin and α-MSH [11, 12]. These hormones are released to the circulation [13], where they may have peripheral actions on metabolic tissues. The melanocortin system includes in total five G-protein coupled receptors (family A) defined as MC1R-MC5R. The MCRs are mostly described for their signaling through coupling to Gs, which increases intracellular adenylate cyclase activity, cAMP and activates protein kinase A (PKA). Peripheral MCRs were initially examined for their effects on pigmentation and adrenal function, which are mediated by MC1R and MC2R respectively. However, the melanocortin system also has other peripheral functions such as in white adipose tissue, where it regulates proliferation (MC1R) [14], leptin release (MC4R and MC5R) [2, 15] and lipolysis (MC2R and MC5R) [16–18]. Moreover, peripheral MCRs regulate thermogenesis in brown adipose tissue (MC2R and MC5R) [19], exocrine gland secretion (MC5R) [20] as well as anti-inflammatory effects (MC1R and MC3R) [21, 22]. Peripheral MCR-regulation of muscle metabolism is a rather unexplored field but it has previously been reported that α-MSH increases fatty acid oxidation in skeletal muscle likely mediated by MC5R and downstream activation of cAMP, PKA, AMPK and ACC [23].

Insulin and exercise/contraction are the two most physiologically relevant stimuli of glucose transport in skeletal muscle [24]. Their effects seem to be additive, since a combination of contraction and insulin causes a greater glucose uptake compared to either stimulus alone [25]. Researchers have pursued the downstream targets of insulin and contraction-mediated glucose uptake, which could operate as a link to the physiological effect on glucose transporter 4 (GLUT4) translocation. TBC1 domain (TBC1D) family member 4 (also termed Akt substrate of 160 kDa (AS160)) as well as TBC1D1 have been suggested to act as downstream mediators of insulin (Akt)and contraction (AMPK)stimulated glucose uptake respectively [26, 27]. TBC1D4 and TBC1D1 are functional Rab-GTPase-activating proteins, which inhibit GLUT4 translocation by keeping target rab proteins inactive [28, 29]. In the phosphorylated state TBC1D4 and TBC1D1 dissociate from GLUT4 storage vesicles, which facilitate GLUT4 translocation and glucose uptake. Hence, TBC1D4/1 can be characterized as intracellular brakes on GLUT4 translocation. Phosphorylation of TBC1D1 S237 and T596 are suggested to be pivotal in terms of GLUT4 translocation in murine muscle [27, 30]. Also, several phosphorylation sites on TBC1D4 have been associated with GLUT4 translocation in murine muscle including T642 [31, 32].

In this study, we examined the molecular signaling pathway induced by α-MSH during stimulation of glucose uptake in murine muscle. As AMPK is known as an essential mediator of glucose uptake, we characterized the downstream signaling pathway of AMPK during α-MSH stimulation in differentiated L6 myotubes and soleus muscle explants. Furthermore, we assessed the effect of α-MSH on glucose uptake in soleus and extensor digitorum longus (EDL) muscle explants from wild type (WT) and α_2_-AMPK kinase-dead (KD) mice. Lastly, we investigated the phosphorylation pattern of TBC1D1 and TBC1D4 and the translocation of GLUT4 in response to α-MSH stimulation. We found that α-MSH significantly induces phosphorylation of TBC1D1 (S237 and T596) independently of upstream PKA and AMPK. We found no evidence to support that α-MSH-stimulated glucose uptake involves phosphorylation of TBC1D4 (T642 and S704). Likewise, α-MSH does not seem to induce GLUT4 translocation in cultured muscle cells.

## Methods

### Test Compounds

α-MSH (Bachem), PKA inhibitor H89 dihydrochloride hydrate (Sigma-Aldrich Co. LLC), human recombinant insulin (Actrapid, Novo Nordisk).

### Materials

Total RNA from soleus muscles was extracted using the Trizol method and RNeasy kit (Qiagen, Germantown, MD). The total RNA was reverse transcribed using SuperScript II reverse transcriptase. Primers and gene expression mastermix from Applied Biosystems were used in real time PCR. Mice used for experiments were purchased from Taconic. [^3^H] 2-deoxyglucose (Perkin Elmer), [^14^C] mannitol (Perkin Elmer) and liquid scintillation (Tri-Carb 2000, Packard Instrument) was used to measure glucose uptake. Bio-Rad equipment was used to cast gels. PVDF membranes and enhanced chemiluminescence from Millipore were used in western blotting.

### Animals

All mice used for in vivo experiements at the August Krogh Centre were bred and housed at the Department of Experimental Medicin (Panum, University of Copenhagen). WT littermates produced from heterohetero breeding were used as controls for the α_2_-kinase deficient (KD) mice. Mice were maintained on a 12:12-h light-dark cycle and received standard rodent chow and water *ad libitum*. The AMPK KD mouse model has previously been described by Mu et al. [33].The muscle stimulation experiments were conducted on 4 separate days: experiment day 1 (WT [n=4], AMPK KD [n=4]), experiment day 2 (WT [n=6], AMPK KD [n=2]), experiment day 3 (WT [n=7], AMPK KD [n=8]) and experiment day 4 (WT n=8). α-MSH-stimulation of glucose uptake obtained in WT mice from each of the 4 experiment days were pooled in Fig 3A. Data generated in the H89 experiment (Fig 6A) are only obtained from experiment day 4. At Monash University, from 5-6 wk of age C57BL/6J mice (Monash Animal Services) were fed a regular rodent diet (Specialty Feeds) or a HFD (SF04-001; Specialty Feeds) for 20 weeks. Mice were housed (5/cage) in a controlled environment and food as well as water was available *ad libitum*. During each experiment, animals were individually caged. Body weights were measured weekly. Euthanasia: Mice were euthanized by intraperitoneal injection of pentobarbitone sodium anaesthetic (6 mg/100 g body weight) under isofluorine anaesthesia.

### Incubation of isolated muscles

EDL and soleus were quickly removed from anesthetized fed mice (females, 18-23 g) and incubated in Krebs Ringer Buffer (KRB) (117 mmol/l NaCl, 4.7 mmol/l KCl, 2.5 mmol/l CaCl_2_, 1.2 mmol/l KH2PO4, 1.2 mmol/l MgSO4, and 24.6 mmol/l NaHCO3 with addition of 8 mmol/l mannitol, 2 mmol/l pyruvate, and 0.1% BSA, pH = 7.4) as previously described [34, 35]. Muscle explants were suspended at resting tension in Multi Myograph system incubation-chambers (Danish Myo-Technology, Aarhus, DK) at 30°C and oxygenated with a gas containing 95% O_2_ and 5% CO_2_. After a 10 min pre-incubation, muscles were either stimulated with KRB + α-MSH (100 nM) or KRB + DMSO (vehicle; 0.0025%) for 30 minutes. Glucose uptake was measured during the last 10 min of the 30 min period by adding labelled [^3^H]-2-deoxyglucose (1 mmol/l, 0.056 MBq/ml) and [14C]-mannitol (7 mmol/l, 0.0167 MBq/ml) to the incubation medium. After stimulation, muscles were harvested and snap frozen in liquid nitrogen and stored at -80°C for later processing. Dissected muscle was incubated pairwise; i.e., from the same animal, one muscle was used for stimulation, whereas as the contralateral muscle was used as control (basal/rest). At the Monash University, mice (males) were fasted overnight and soleus muscles were dissected tendon to tendon from anaesthetised mice (2-3% isoflurane gas). Muscles were preincubated for 30 min with warmed (30°C), pre-gassed (95%O_2_- 5%CO_2_, pH 7.4), modified Krebs-Henseleit buffer supplemented with 2 mmol/l sodium pyruvate, 8 mmol/l mannitol, and 0.1% wt/vol BSA and were then incubated with or without 10 nM insulin, 100 nM α-MSH or α-MSH plus insulin (100 nM + 10 nM) for 20 min. Glucose uptake was assessed for 10 min using 2-deoxy-D-[2,6-^3^H]glucose (1 mmol/l, 0.5 μCi/ml) and 1 mM D-[14C]mannitol (0.45 mCi/ml) in the presence or absence of 10 nM insulin. Radioactivity was measured in muscle lysates by liquid scintillation counting.

### Preparation of muscle lysate

Intact muscles were homogenized in 400 ml ice-cold lysis buffer [10% glycerol, 20 mM sodium pyrophosphate, 1% NP-40, 2 mM PMSF, 150 mM sodium chloride, 50 mM HEPES, 20 mM β-glycerophosphate, 10 mM sodium fluoride, 1 mM EDTA, 1 mM EGTA, 10 g/ml aprotinin, 3 mM benzamidine, 10 g/ml leupeptin, and 2 mM sodium orthovanadate (pH 7.5)] for 2 x 1 minute using a tissuelyser (TissueLyser II, Qiagen, Germany). Homogenates were rotated end over end for 1 h at 4°C and subsequently centrifuged at 16,000 *g* at 4°C for 20 min. Supernatants were collected and snap-frozen in liquid nitrogen and stored at -80°C for later analyses. Total protein concentrations were determined using BCA protein assay (Pierce Biotechnology Inc., Rockford, IL).

### Differentiation of L6 myotubes and preparation of lysate

L6 myoblasts [36] were thawed and seeded with DMEM (+10% FBS, 1% pen/strep). At 70% of confluence, cells were trypsinized and seeded in 12 wells plates. One day after reaching 100% confluence, differentiation was started by changing growth media to low glucose DMEM + 2% horse serum. Media was replaced every second day during 9 days. Approximately 40% of the cells were differentiated into myotubes on the day of the experiment. Medium was replaced by fresh serum-free low glucose medium (1h) and cells were stimulated with vehicle or 100 nM α-MSH for 20 minutes. Cells were washed 3 times with PBS at 37° C before adding solubilization buffer (PBS + 0.1% Triton-X). Homogenates were centrifuged at 16.000 *g* at 4°C for 20 min. Supernatants were collected and stored at -80°C for later analyses. Total protein concentrations were analyzed using BCA protein assay (Pierce Biotechnology Inc., Rockford, IL).

### Western blotting

Muscle lysates were adjusted to equal protein concentration and heated in Laemmli buffer (5 min, 96°C) as previously described (35). Samples (20 g) were separated by SDS-PAGE loaded on self-cast 5 or 8% acrylamide gels and transferred to PVDF membranes. Membranes were blocked (1 h at room temperature) in washing buffer (10 mmol/l Tris-base, 150 mmol/l NaCl, and 0.25% Tween 20) containing 2% low-fat milk protein. L6 myotube lysates were adjusted to equal protein concentration and heated in NuPAGE LDS Sample buffer + 10 X DTT NuPAGE Sample Reducing Agent (5 min, 96°C). Samples (20 g) were loaded on pre-cast 10% Bis-Tris gels and transferred to PVDF membranes. Membranes were blocked (1 h at room temperature) in blocking buffer (Thermo Scientific). Membranes were incubated with primary antibodies (1:1000) overnight at 4°C, followed by incubation (1 h at room temperature) with appropriate secondary antibody (1:5000) (polyclonal goat anti-rabbit or rabbit anti-sheep, DakoCytomation). At the University of Copenhagen, bands were visualized using a ChemiDoc (MPsystem, BioRad) and quantified using Image Lab software. At Monash University, bands were analysed using AlphaView Software from ProteinSimple. Primary antibodies: TBC1D1 (#5929, Cell Signaling Technology), TBC1D1 phospho (p) 237 (Cat. No. 07-2268, Millipore), TBC1D1 p596 (#6927, Cell signalling), TBC1D1 p700 (#6929, Cell signalling), TBC1D4 p642 (#3028 P1, Symansis), TBC1D4 p704 (kindly provided by Dr. L.J. Goodyear, Joslin Diabetes Center, Harvard Medical School, Boston, MA), TBC1D4 protein (Cat. No. 07-741, Millipore), Akt p473 (#9215, Cell Signaling Technology), Akt 2 (#3063, Cell Signaling Technology, ACC total (HRP conjugated streptavidin, DakoCytomation), ACC p222 (#JBC1900509, Millipore), AMPK2 (#SC-19131 Santa Cruz) and AMPK p172 (#2535, Cell Signaling Technology).

### Glucose tolerance test (GTT) and Insulin Tolerance Test (ITT)

**Fig 1:**
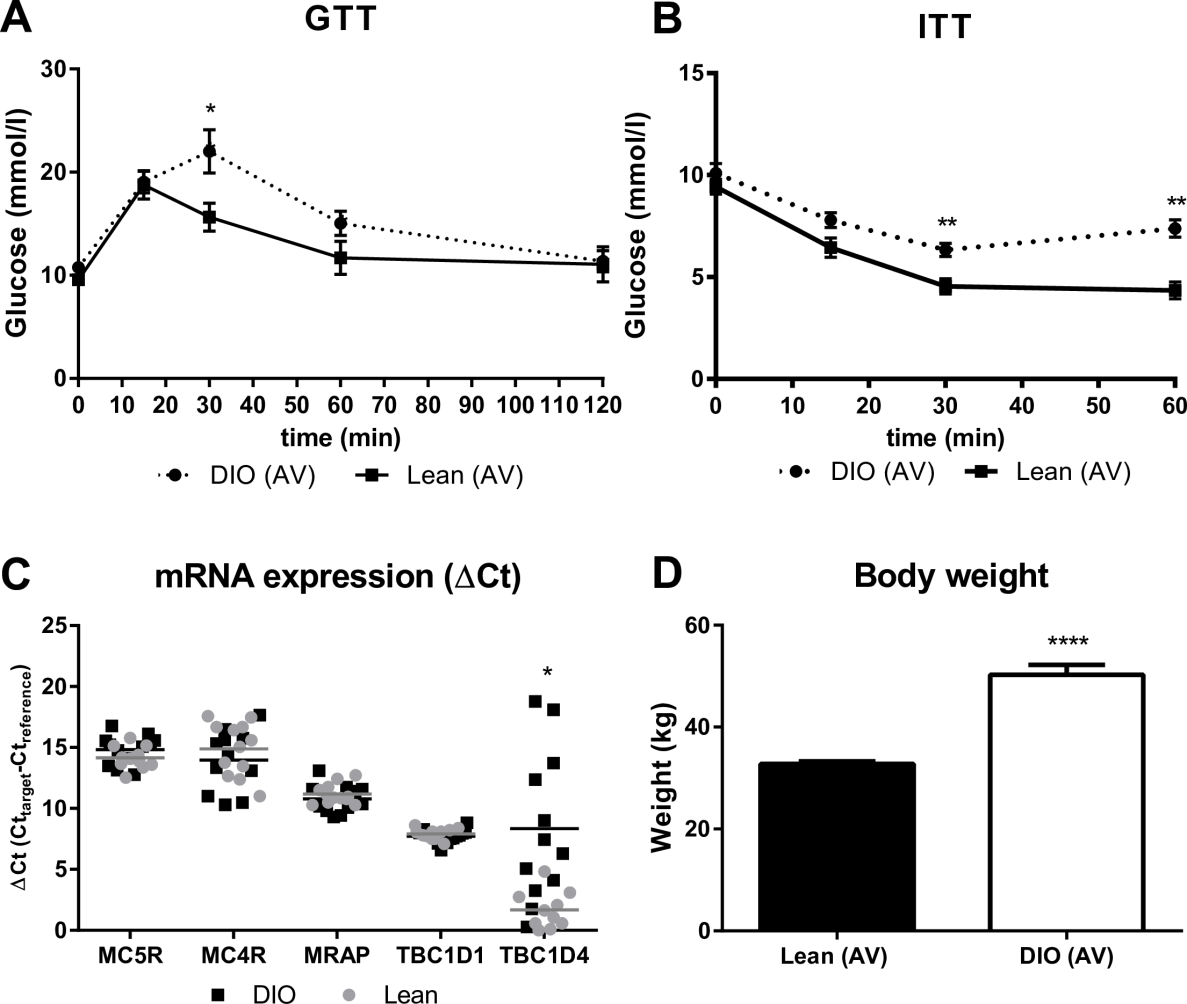
Relative mRNA expression of MC5R, MC4R, MRAP, TBC1D1 and TBC1D4 in soleus muscle dissected from lean and DIO mice. RNA was extracted from lean and DIO mice (n=12), which were characterized as obese and insulin resistant prior to the dissection of soleus by body weight, GTT and ITT (n=6) (A, B and D). cDNA was synthesized and relative mRNA levels were determined by real time PCR (C). Data are shown as mean ± SEM. Two-tailed t-test calculated from normal distributed Ct-levels; p-value (*p 0.05, **p 0.01, ***p 0.001).

**Fig 2:**
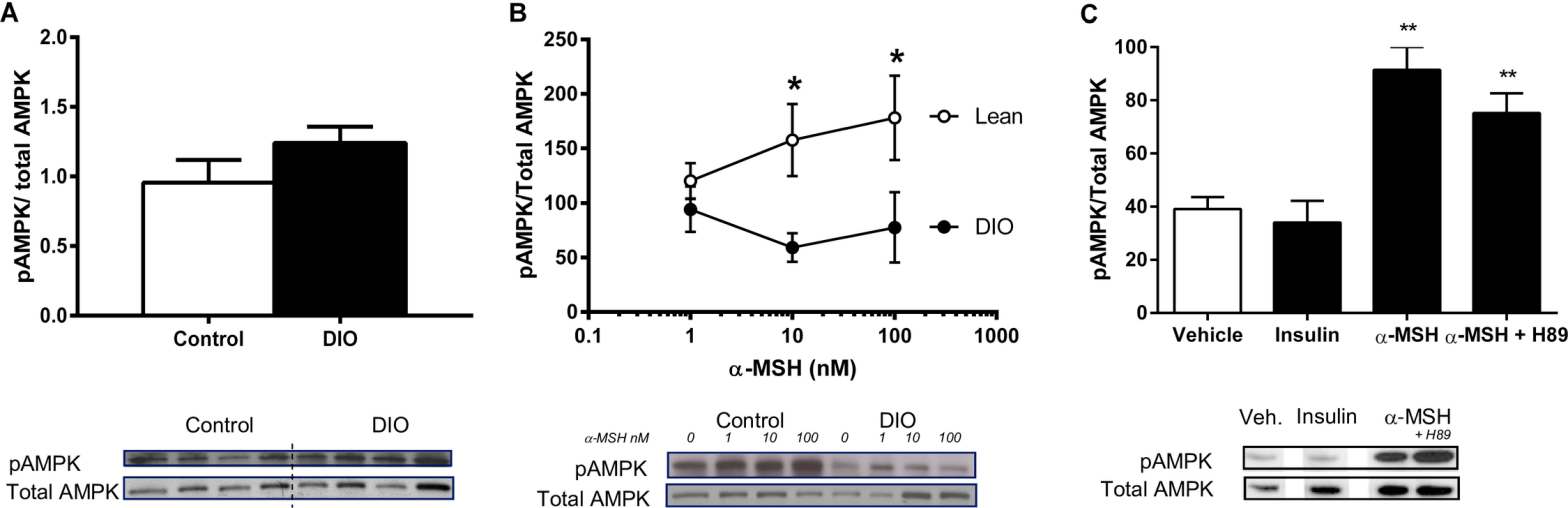
α-MSH induced phosphorylation of AMPK in differentiated L6 myotubes and in soleus explants dissected from lean and diet-induced obese (DIO) mice. A: Phosphorylation of AMPK in lean and DIO mice in basal conditions in soleus muscle explants (n=4). 2.B: α-MSH-phosphorylation of AMPK in lean and DIO soleus muscle explants (n=6). 2.C: Phosphorylation of AMPK after α-MSH-stimulation (100 nM) in differentiated L6 myotubes (n=4). AMPK phosphorylation is normalized to total AMPK. Unpaired one-tailed t-test was used to calculate statistical significance (*p < 0.05, **p < 0.01, ***p < 0.001 vs. vehicle)

After a 4-hour fast samples were obtained from tail vein bleeds from control and DIO mice. Blood glucose was measured using a glucometer (Accu-check; Roche Diagnostic Corp) at 0, 15, 30, 60, and 120 minutes after an ip injection of glucose (1 mg/g). Insulin Tolerance Test (ITT) was also measured after a 4-hour fast from the tail vein bleeds in control and DIO mice at t= 0, 15, 30 and 60 minutes after an ip injection of human insulin (1.0 U/kg; Lilly, Indianapolis, IN). Previous inhouse studies have shown that just ~70% of the mice fed on HFD were glucose intolerant and insulin resistant. For this reason we performed GTT and ITT to select glucose intolerant and insulin resistant mice. Selection of mice was only used in experiments shown in Figs 1 and 2, where the direct effect in DIO mice specifically was examined.

### RNA extraction and cDNA synthesis

Dissected soleus explants were used for RNA extraction and subsequent real time PCR. Samples were collected from 12 lean and 12 diet induced obese (DIO) mice. Mice were characterized as obese and insulin resistant prior to the dissection of tissue by whole body weight, intraperitoneal glucose (GTT) and insulin tolerance (ITT) tests as routinely done in our laboratory [37]. Quality of RNA was quantified by measuring absorbance at 260 nm and 280 nm (ratio should be 1.8 or higher). cDNA synthesis reactions were incubated 5 min at 25°C, 30 min at 42°C and 5 min at 85°C.

### Real time PCR

Gene expression assays for MC4R (Mm00457483_s1), MC5R (Mm00442970_m1), TBC1D1 (Mm00497989_m1), TBC1D4 (Mm00557659_m1) and MRAP (Mm00547149_m1) were purchased from Applied Biosystems and standard curves for each primer set were initially run to establish a suitable cDNA dilution factor. Each reaction was prepared by mixing 5 μl qPCR mastermix, 0.5 μl of primer/probe and H_2_O (up to 8 μl). Synthesized cDNA was diluted 1:10, after which 2 μl of diluted cDNA in total reaction volume of 10 μl were added to a 96 well plate. All samples were run in triplicates and plates were centrifuged (30 seconds/1000 rpm) prior to real time PCR. GAPDH was chosen as reference gene.

### GLUT4 translocation assay

The rat L6 muscle cell line stably overexpressing exofacially myc-epitope tagged GLUT4 has previously been described [38]. L6 GLUT4myc myoblasts were seeded into 96-well plates (100 l) and grown to confluence in MEM with 10% FBS after which the serum was lowered to 2% FBS to initiate myotube differentiation for 6 days. The medium was changed every 2 days. On day 6 post-differentiation, the L6 myotubes were serum starved for 1.5 h after which DMSO alone, increasing doses of α-MSH (0.1-500 nmol/l) or insulin were added and incubated for 30 min. Cells were then washed in ice-cold PBS, fixed in 3% paraformaldehyde and blocked in 5% goat serum before incubation with anti-myc antibody (Sigma C3956 1:500) for 45 min, then anti-rabbit HRP secondary antibody (DAKO P0448 1:1000) for 30 min before colorimetric absorbance-detection at 495 nm using OPD (Sigma P5412). In one well, primary antibody was omitted to measure the background absorbance for subtraction. The mean of 11-12 wells/condition/plate was taken as an n=1 and the experiment repeated 3 times.

### Statistical analysis

The use of radiolabeled 2-DG is a widely used method to establish glucose uptake in muscle explants/cultures and have previously been described [39, 40]. Western blot bands normalized to the vehicle group (CTR) and analyzed in GraphPad Prism. Real-time quantization of target mRNA was normalized to GAPDH. Data were obtained as threshold cycle (Ct) values and relative gene expression was calculated using the Ct method as described in the User Bulletin No. 2, 1997 (ABI, 7700 Sequence Detection System). Data are presented as Ct values (Ct_(target gene)_ – Ct_(reference gene)_), which are also used for calculations of significance (normal distributed). Students t-test was used to calculate statistical significance between 2 groups. 2-way repeated measures (RM) ANOVA (Student Newman Keuls post hoc test) was used to calculate a statistical significant effect of α-MSH or insulin, when comparing more than 2 groups (*p < 0.05, **p < 0.01, ***p < 0.001). ^#^ indicates a significant effect of genotype (^#^p < 0.05, ^##^p < 0.01, ^###^p < 0.001).

### Ethics statement

All experiments performed at the August Krogh Centre were approved by the Danish Animal Experimental Inspectorate. At Monash University, all procedures were performed in accordance with the guidelines and approval of the Monash University Animal Ethics Committee.

## Results

### mRNA expression levels of TBC1D1, TBC1D4, MC5R, MC4R and MRAP in soleus muscle dissected from lean vs. DIO mice

Previous studies have shown that mutations in MCRs cause obesity. Intriguingly, it has not been investigated whether obesity induces a change in the expression of muscle MCRs. For this reason we initially characterized the expression profile of target genes in soleus muscle of lean vs. DIO mice. RNA was extracted from lean and DIO mice, which were characterized as obese and insulin resistant (Fig 1A, 1B and 1D). We found that TBC1D4 mRNA was significantly enriched (5.5 fold) in soleus muscle dissected from DIO mice compared to lean (average of absolute Ct-values were 31.9 vs. 33.5) (Fig 1C). To our knowledge this result has not previously been described. TBC1D1, MC4R, MC5R and MCR accessory protein (MRAP) mRNA were expressed at similar levels in DIO mice compared to lean.

### α-MSH-stimulated phosphorylation of AMPK in differentiated L6 myotubes and in soleus muscle dissected from lean vs. DIO mice

AMPK is a key integrator of hormones and nutrient signals that regulates glucose transport and fatty acid oxidation in muscle. We examined the capacity of α-MSH to stimulate phosphorylation of AMPK T172 in lean vs. DIO *soleous*. Phosphorylation of AMPK was not different under basal conditions (Fig 2A). However, treatment with α-MSH caused a dose-dependent phosphorylation of AMPK in muscle obtained from lean mice only (Fig 2B). MCRs are described to signal through coupling to Gs, which increases intracellular adenylate cyclase, cAMP and activate protein kinase A (PKA). Thus, we examined the effect of α-MSH on AMPK phosphorylation in the presence of PKA inhibitor H89. In differentiated L6 myotubes we found that H89 did not prevent α-MSH induced phosphorylation of AMPK (Fig 2C). This implies that α-MSH induces phosphorylation of AMPK T172 through a mechanism independent of PKA.

### α-MSH-stimulated glucose uptake in WT and AMPK KD mice

**Fig 3:**
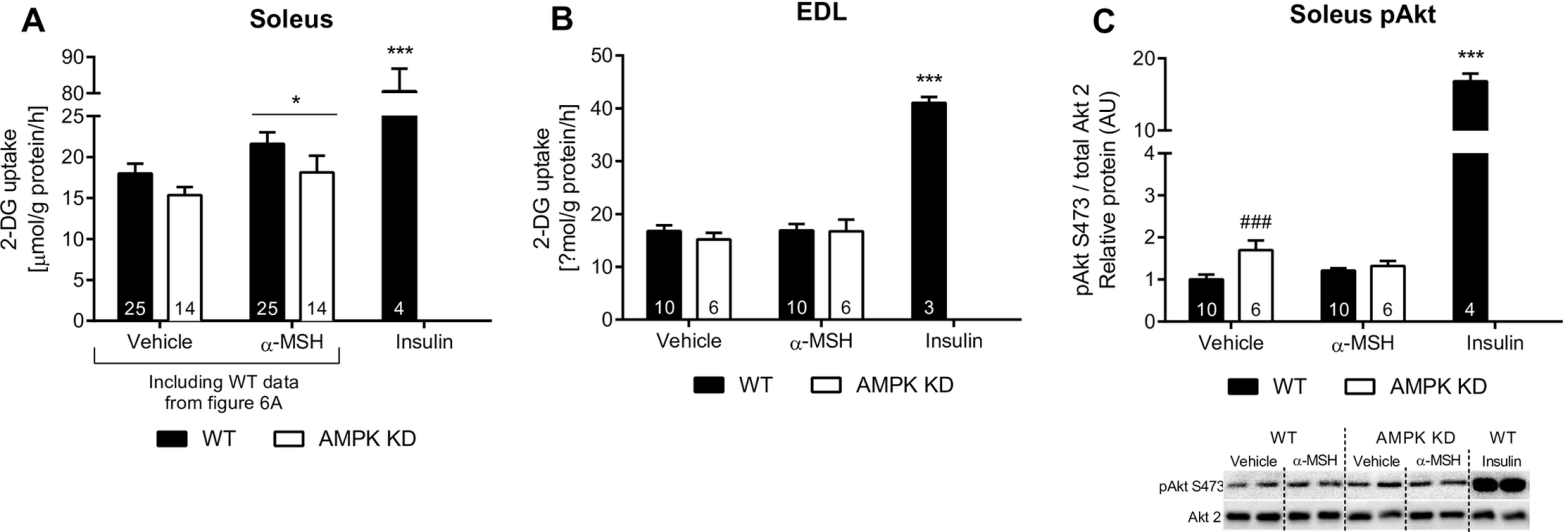
α-MSH stimulated 2-Deoxy Glucose uptake in soleus and extensor digitorum longus (EDL) muscle explants. 3.A: soleus explants from WT and AMPK KD mice were stimulated with α-MSH (100 nM). Data is presented as the mean ± SEM of pooled data from a series of experiments (see individual bars). 3.B: EDL explants from WT and AMPK KD mice were stimulated with α-MSH (100 nM). 3.C: Phosphorylation of Akt was measured in soleus explants after α-MSH-stimulation (100 nM). Data is presented as the mean ± SEM. 2-way RM ANOVA was used to calculate statistical significance (*p < 0.05, **p < 0.01, ***p < 0.001 vs. vehicle). ^#^ indicates a significant effect of genotype (^#^p < 0.05, ^##^p < 0.01, ^###^p < 0.001).

To investigate wheather the phosphorylation of AMPK is necessary for α-MSH induced glucose uptake and signaling, we took advantage of the AMPK KD mouse model [33]. α-MSH significantly increased glucose uptake by ~20% in soleus muscle from both WT mice and AMPK mice (Fig 3A), indicating that α-MSH increases muscle glucose uptake independently of AMPK. Notably, we found no effect of α-MSH on glucose uptake in EDL (Fig 3B). For this reason only soleus muscle was used for further examination of α-MSH-induced signaling. As previously reported by An et al. [22], we found that α-MSH induces phosphorylation of AMPK T172 and downstream target ACC S212 in WT animals (S1 Fig). In order to exclude the possibility that α-MSH elicits its effect on glucose uptake by potentiation of insulin-like signaling, we next examined the effect of α-MSH on Akt phosphorylation. We observed that α-MSH did not increase phosphorylation of Akt T473in soleus muscle (Fig 3C).

### α-MSH-stimulated phosphorylation of TBC1D1 and TBC1D4 in soleus muscle

**Fig 4:**
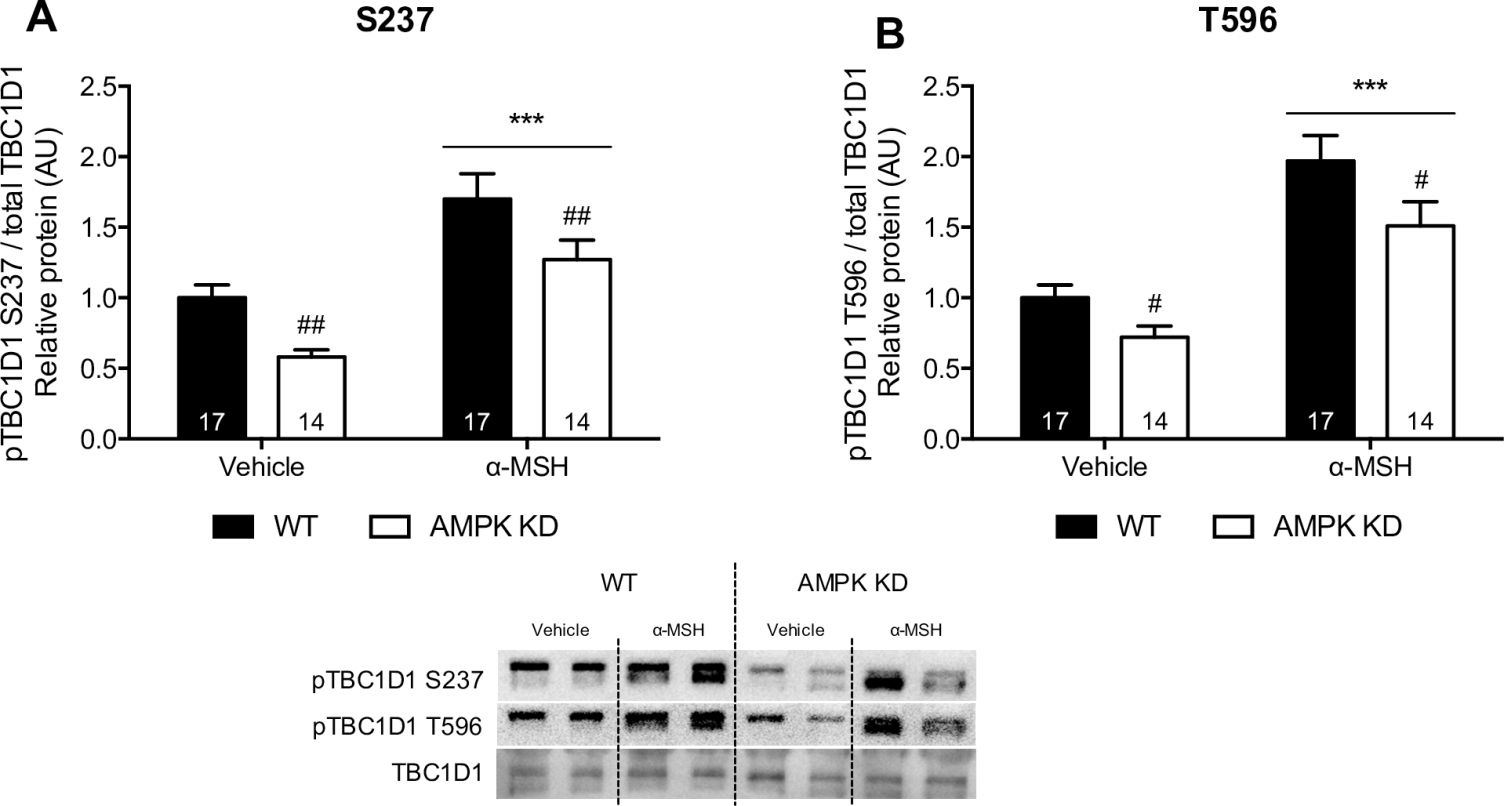
α-MSH (100 nM) stimulated TBC1D1 S237 and T596 phosphorylation in WT and AMPK KD mice. TBC1D1 S237 and T596 phosphorylation sites were measured in soleus muscle using WB as described (n indicated in the individual bars). Phosphorylation of TBC1D1 S237 and T596 is normalized to total TBC1D1. Findings are shown as representative immunoblots and pooled data is quantified in bar graphs as arbitrary units. 2-way RM ANOVA was used to calculate statistical significance (*p < 0.05, **p < 0.01, ***p < 0.001 vs. vehicle). ^#^ indicates a significant effect of genotype (^#^p < 0.05, ^##^p < 0.01, ^###^p < 0.001).

**Fig 5:**
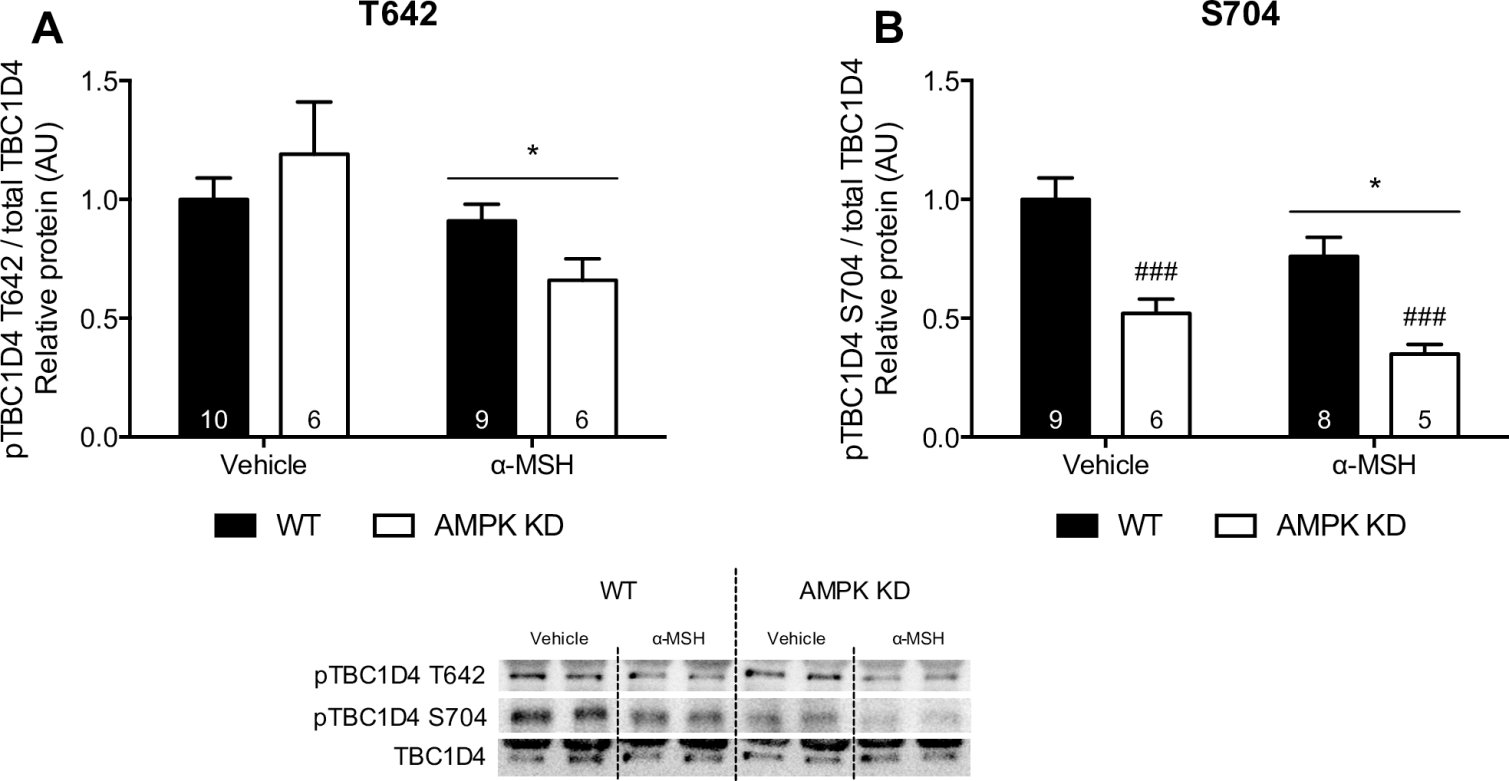
α-MSH (100 nM) stimulated TBC1D4 T642 and S704 phosphorylation in WT and AMPK KD mice. Phosphorylation of TBC1D4 was measured in soleus muscle using WB as described (n indicated in the individual bars). TBC1D4 T642 **and S704** phosphorylation is normalized to total TBC1D4. Findings are shown as a representative immunoblot and pooled data is quantified in bar graphs as arbitrary units. 2-way RM ANOVA was used to calculate statistical significance (*p < 0.05, **p < 0.01, ***p < 0.001 vs. vehicle). ^#^ indicates a significant effect of genotype (^#^p < 0.05, ^##^p < 0.01, ^###^p < 0.001).

To further disclose the downstream signaling pathway induced by α-MSH, we measured phosphorylation of Rab-GTPase-activating protein TBC1D1 and TBC1D4 in WT and in AMPK KD mice. We hypothesized that α-MSH induces phosphorylation of TBC1D1, which increases GLUT4 translocation and glucose uptake. We found that α-MSH significantly stimulates phosphorylation of TBC1D1 S237 and T596 in WT and in AMPK KD mouse soleus muscle (Fig 4A and 4B). This suggests that phosphorylation of TBC1D1 in response to α-MSH is mediated by an AMPK-independent mechanism. Phosphorylation of TBC1D4 was also studied as a potential downstream mediator of α-MSH-induced glucose uptake. However, we found no increase in phosphorylation of TBC1D4 T642 and S704 after α-MSH-stimulation in either WT or AMPK KD mice (Fig 5A and 5B). Notably, we found a significant reduction in TBC1D4 T642 and S704 phosphorylation after α-MSH stimulation in both genotypes.

### α-MSH-stimulated glucose uptake and TBC1D1 phosphorylation in soleus muscle with or without treatment with PKA-inhibitor H89

**Fig 6:**
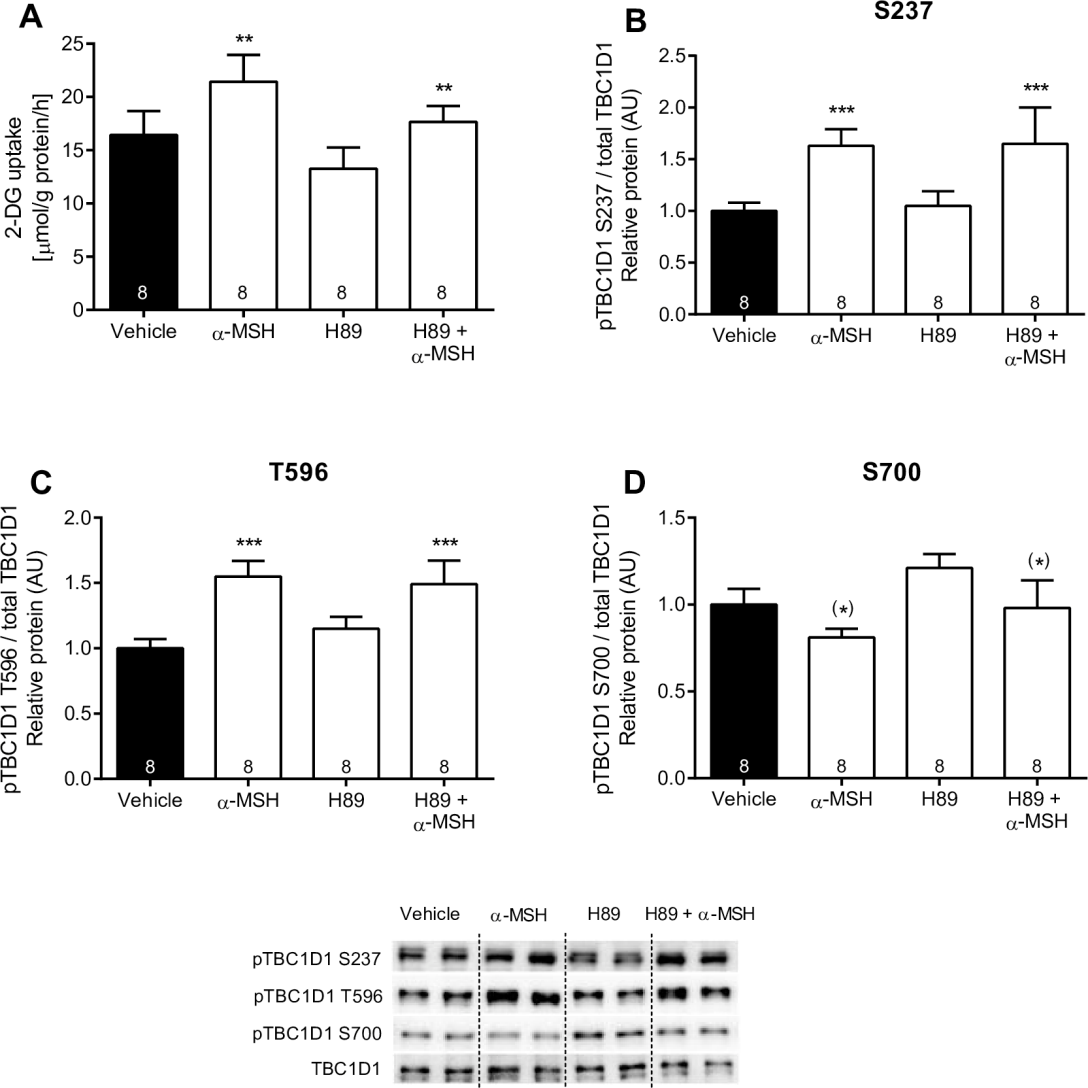
α-MSH (100 nM) stimulated 2-Deoxy Glucose uptake and TBC1D1 S237, T596 and S700 phosphorylation +/- H89 in dissected soleus explants from WT mice. A: Soleus muscle was dissected, stimulated and 2-DG was measured as described (n indicated in the individual bars). B: Phosphorylation of TBC1D1 was measured in soleus muscle using WB as described. TBC1D1 S237, T596 and S700 phosphorylation is normalized to total TBC1D1. Findings are shown as a representative immunoblot and pooled data quantified in bar graphs as arbitrary units. 2-way RM ANOVA was used to calculate statistical significance (*p < 0.05, **p < 0.01, ***p < 0.001 vs. vehicle). ^#^ indicates a significant effect of genotype (^#^p < 0.05, ^##^p < 0.01, ^###^p < 0.001). Data generated in the experiment are only obtained from experiment day 4.

Since PKA is an established mediator of MCR signaling, we investigated α-MSH-induced glucose uptake and TBC1D1 phosphorylation in the presence of PKA inhibitor H89. α-MSH-induced glucose uptake was not significantly affected by H89 indicating that PKA does not mediate the effect. Likewise, H89 did not decrease α-MSH-mediated phosphorylation of TBC1D1 S237 and T596 (Fig 6B and 6C). We found that α-MSH induced a minor reduction in phosphorylation of TBC1D1 S700. Collectively, the results suggest that phosphorylation of TBC1D1 is not directly regulated by MC5R-PKA signaling

### α-MSH-stimulated GLUT4-translocation in differentiated L6 myotubes

**Fig 7:**
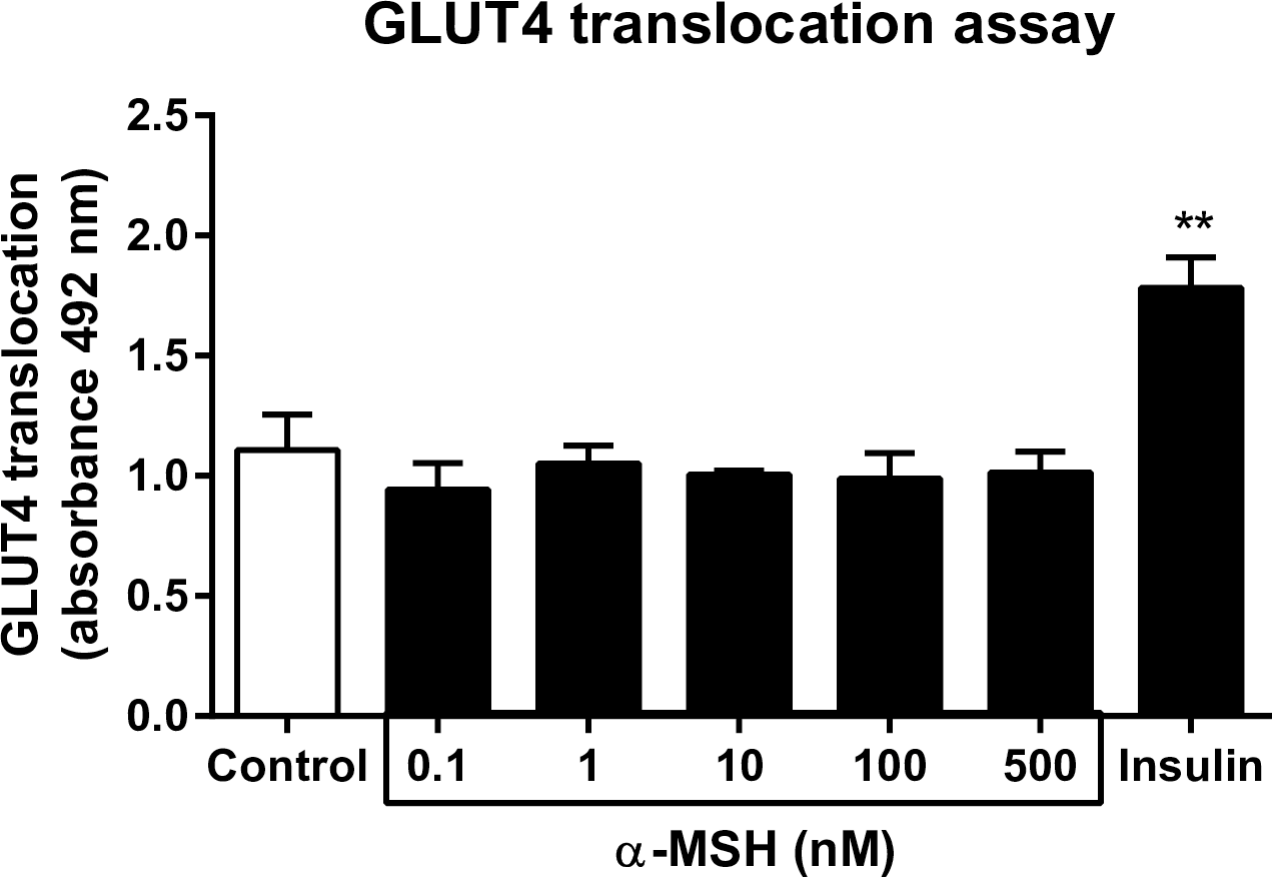
α-MSH stimulated GLUT4 translocation in differentiated L6 myotubes (n=3). Differentiated L6 myotubes were serum starved for 1½ h after which DMSO alone, increasing doses of α-MSH (0.1-500 nM) or insulin were added for 30 minutes. Data is presented as mean ± SEM. 2-way RM ANOVA was used to calculate statistical significance (*p < 0.05, **p < 0.01, ***p < 0.001 vs. CTR).

To investigate whether α-MSH-stimulated glucose uptake is mediated by a change in GLUT4 translocation we measured translocation of GLUT4 after α-MSH stimulation in differentiated L6 myotubes. Insulin significantly increased GLUT4 translocation. However no effect of α-MSH was observed (0.1-500 nM) (Fig 7).

## Discussion

This study was conducted to gain insight into the molecular signaling induced by α-MSH during glucose uptake in skeletal muscle. cAMP and PKA are known downstream mediators of MCR signaling. Thus, we hypothesised that α-MSH-stimulated glucose uptake involves PKA, AMPK and potentially Rab-GTPase-activating protein TBC1D1 or TBC1D4. Here we show that α-MSH stimulates glucose uptake in mouse skeletal muscle, which no not seem to involve PKA. Furthermore, our results suggest that α-MSH induces phosphorylation of AMPK and TBC1D1. However these effects appear to occur independently of PKA- and PKA/AMPK-signaling, respectively. Collectively, these observations represent significant findings of novel signaling for melanocortin peptides and their receptors not previously described.

**Fig 8:**
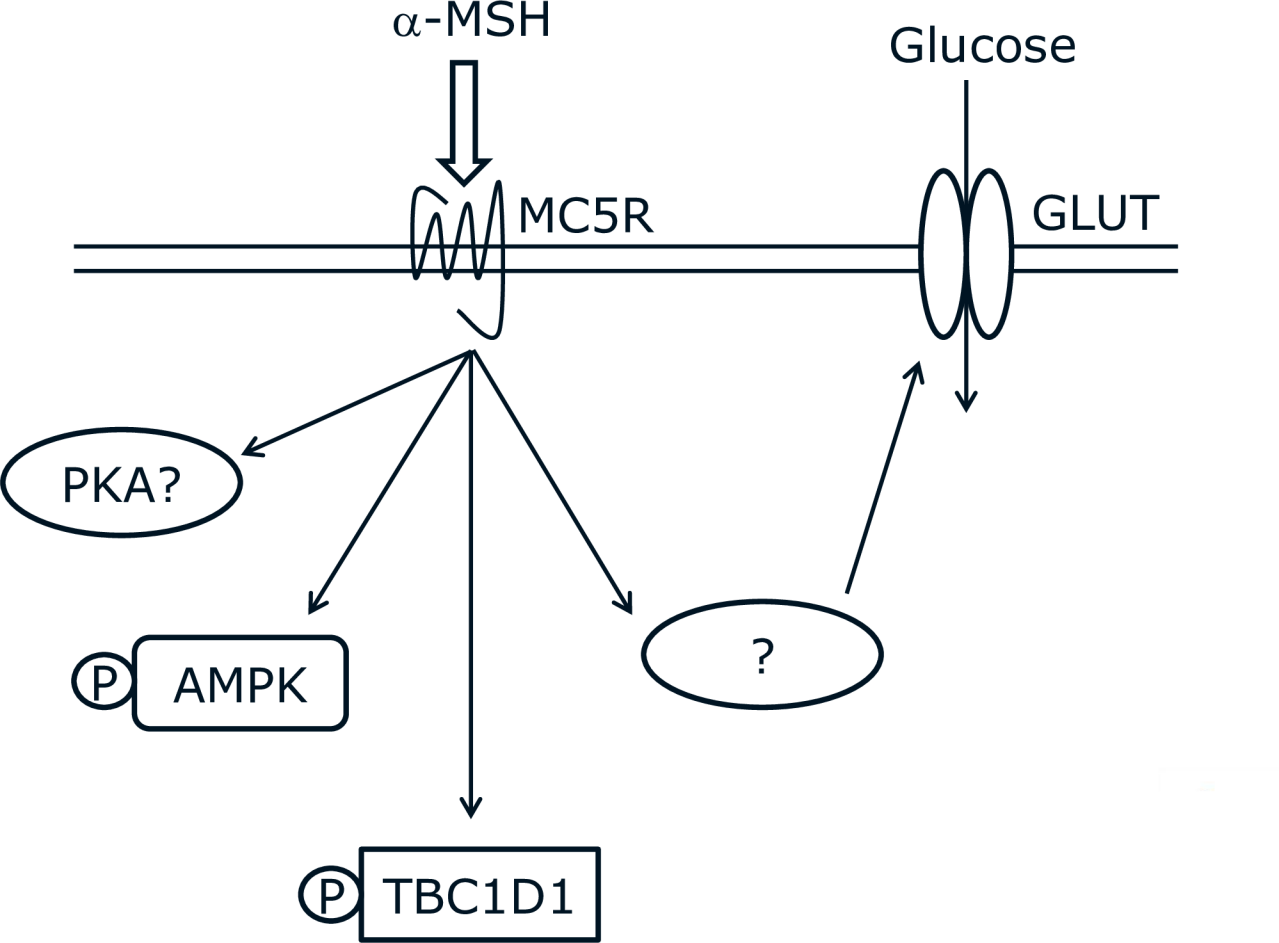
Overview of cellular signaling induced by α-MSH in skeletal muscle. α-MSH stimulates glucose uptake by an unknown mechanism, which acts independently of AMPK, TBC1D1 and GLUT4 translocation. α-MSH stimulates phosphorylation of TBC1D1 independently of PKA and AMPK. α-MSH stimulates phosphorylation of AMPK independently of PKA.

Based on glucose uptake measurements in the AMPK KD mouse model, AMPK does not seem to be involved in the ability of α-MSH to stimulate muscle glucose uptake.Evidence suggests that AMPK stimulates muscle glucose uptake by increasing the amount of GLUT4 in the cell surface membrane [41]. However, we did not find an effect of α-MSH on GLUT4 translocation further supporting the notion that α-MSH stimulates glucose uptake independently of AMPK. Although, it seems unlikely that α-MSH induces phosphorylation of AMPK T172 without facilitating improvements in GLUT4 translocation, it may be explained by the selective action of α-MSH towards specific AMPK heterotrimeric complexes. Such selectivity has been demonstrated for the unspecific AMPK activator PT-1, which increases phosphorylation of AMPK T172 without affecting glucose uptake in mouse soleus and EDL [41, 42]. Regarding TBC1D4, we found a significant reduction in T642 and S704 phosphorylation after α-MSH stimulation in WT and AMPK KD mice. Based on a previous mutation study [31] this indicates that α-MSH-induced glucose uptake occurs independently of TBC1D4-mediated GLUT4 translocation. Similar to TBC1D4, it has been suggested that TBC1D1 controls GLUT4 translocation and downstream glucose uptake [43]. Our results suggest that α-MSH stimulates glucose uptake independently AMPK, TBC1D1 and TBC1D4. This is in line with the observation that GLUT4 translocation is not involved in α-MSH-stimulated glucose uptake (Fig 8). It would be highly relevant to measure α-MSH-stimulated glucose uptake in a TBC1D1-deficient model, which unfortunately was not possible in this study.

It seems likely that other kinases besides PKA are activated after α-MSH stimulation, which potentially phosphorylates TCB1D1. This may explain the increased phosphorylation of TBC1D1 S237 and T596 when co-incubating α-MSH with PKA-inhibitor H89.

Like MCRs, -adrenergic repectors are classically described for their signaling through coupling to Gs, which increases intracellular adenylate cyclase activity, cAMP and activates PKA. Interestingly, adrenaline has also been shown to increase glucose uptake independently of PKA in smooth muscle through -adrenergic repectors [44].

α-MSH induces an increase in TBC1D1 S237 and T596 phosphoryaltion in muscle from AMPK KD mice revealing a previously unregcognized regulation of TBC1D1. Since Akt phosphorylation did not change in response to α-MSH an increase in TBC1D1 phosphoryaltion seems not to be mediated by elevated Akt activity. Thus, one or several protein kinases different from AMPK and Akt appear to regulate phosphorylation of TBC1D1 in response to α-MSH. Alternatively, α-MSH may inhibit the activity of protein phosphatases thereby increasing the overall phosphorylation of TBC1D1. Arguing against this, we found that α-MSH did not increase phosphorylation of TBC1D1 S700. The molecular mechanisms accounting for α-MSH regulation of TBC1D1 phosphorylation needs further clarification.

We found that α-MSH phosphorylates AMPK T172 in muscle explants isolated from lean mice, which is lost DIO mice. However, MC4-5R mRNA levels were equal in muscle from lean vs. DIO mice indicating that the difference is not caused by changes in receptor gene expression. The result that MC4R and MC5R mRNA were similarly expressed in soleus muscle tissue is slightly different than standard PCR published by An et al. stating that MC5R mRNA is substantially higher expressed compared to MC4R mRNA [23].

The signaling pathway disclosed in this study may be involved in metabolic functions other than glucose uptake. TBC1D1 is linked to human obesity and TBC1D1 mutations in lean mouse confer leanness and protects from diet-induced obesity [45]. Expression of TBC1D1 has been identified in skeletal muscle but also in heart, pancreas, colon, kidney and hypothalamus, though at relatively lower expression levels [45]. The MC3R and MC4R are highly expressed in the hypothalamus [11], which raises the possibility that TBC1D1 may execute downstream effects of the melanocortin receptors here as well. Knockdown of TBC1D1 in skeletal muscle cells has been shown to increase fatty acid uptake and oxidation [45]. Hence, the functional end-point of α-MSH and downstream TBC1D1 may likely include fatty acid uptake and oxidation as previously published by An et al. [23]. Notably, TBC1D1 KO mice exhibit normal glucose tolerance, with no difference in body weight compared with WT littermates [46], although these results may be biased on an overall reduction of GLUT4 in TBC1D1 KO mice. Regardless, results obtained in this study support previous data, which propose that the peripheral melanocortin system may be a part of an endocrine circuit that could contribute to the regulation of post-prandial glucose homeostasis. Satiety is induced as a result of central MC4R activation and owing to activation of MC4R coupled to the sympathetic nervous system innervation of liver, muscle, pancreas, brown adipose tissue and white adipose tissue occurs [47–51]. In line with the above, it seems plausible that α-MSH secreted to the circulation may boost central effects on metabolism by inducing glucose uptake in muscle.

Only α-MSH has been tested in mouse soleus muscle explants and this agonist has equal affinity for MC4R and MC5R. Since both MC4R and MC5R mRNA is expressed in mouse soleus muscle we cannot exclude that MC4R also facilitates α-MSH-induced glucose uptake. It would be highly relevant to test MC4R and MC5R selective agonists in order to separate the effect of the receptors. Interestingly, α-MSH did not stimulate GLUT4 translocation in differentiated L6 myotubes. This indicates that the sensitivity of the assay was too low, that GLUTs in the plasma membrane were activated, or that other GLUTs were translocated upon α-MSH stimulation which could explain changes in muscle glucose uptake. It would be interesting to examine translocation of GLUT12 after α-MSH stimulation, as GLUT12 may provide redundancy to the dominant GLUT4 system in muscle [52]. It is estimated that 12% of insulin-induced translocation of glucose transporters in humans are due to GLUT12[52]. In this study, α-MSH-stimulated glucose uptake accounts for ~5% of the maximum insulin-stimulated glucose uptake. Hence, the effect induced by α-MSH is compatible with the GLUT12 capacity. Additional studies are needed to resolve which glucose transporter isoforms are employed during α-MSH-stimulation.

The physiological relevance of melanocortin-induced glucose uptake is difficult to determine. The actual level of circulating endogenous α-MSH is reported to be 11 pmol/l (0.011 nM) calculated as an average of lean and obese patients [53]. This concentration is not comparable with the concentration of α-MSH used in this study. Notably, the physiological concentration of endogenous α-MSH circulating in the human body is below the EC_50_ values for all peripheral MCRs [53], including MC1R in the skin. MC1R is specifically expressed in melanocytes of the skin, where α-MSH stimulation induces synthesis of the brown/black pigment eumelanin [54]. POMC is produced locally in the skin, processed to α-MSH and has a paracrine effect on MC1R. Paracrine stimulation could potentially also take place in other tissues, where POMC and melanocortin receptors are present. Hence, ectopic melanocortin (both α-MSH and ACTH) could stimulate MCRs (MC4/5R) locally in muscle tissue. In addition, a MC4R selective drug is still an attractive anti-obesity target. Peripheral effects on muscle glucose uptake (and fatty acid oxidation) may become relevant, as the therapeutic compound may accumulate in the body.

It may be relevant to consider a combined MC4R/MC5R agonist or a selective MC5R candidate for a therapeutic approach to decrease plasma blood sugar by increasing glucose uptake in muscle.

## Supporting Information

S1 Fig α-MSH (100 nM) stimulated ACC phosphorylation in WT and AMPK KD mice. Phosphorylation of ACC S212 was measured in soleus muscle using western blotting as described. ACC phosphorylation was normalized to total ACC. We found that α-MSH stimulation phosphorylated AMPK in WT animals as previously reported by An et al. [23] (Fig 6). Phosphorylation of AMPK in AMPK KD mice was also identified, however no difference was seen between vehicle and α-MSH-treated explants. AMPK KD mice are not able to phosphorylate ACC, since ACC is a read-out of AMPK activity. We found no increase in ACC-phosphorylation after α-MSH-stimulation in AMPK KD mice, which validates the deficient kinase activity of the AMPK _2_ mutant (Fig 6). It is shown that α-MSH significantly phosphorylates ACC in WT mice proving activity of AMPK. Findings are shown as a representative immunoblot and pooled data are quantified in bar graphs as arbitrary units. 2-way RM ANOVA was used to calculate statistical significance (*p < 0.05, **p < 0.01, ***p < 0.001 vs. vehicle). ^#^ indicates a significant effect of genotype (^#^p < 0.05, ^##^p < 0.01, ^###^p < 0.001).
(TIF)
