# Correction: α-MSH Stimulates Glucose Uptake in Mouse Muscle and Phosphorylates Rab-GTPase-Activating Protein TBC1D1 Independently of AMPK

**DOI:** 10.1371/journal.pone.0161047

**Published:** 2016-08-08

**Authors:** The PLOS ONE Staff

## Notice of Republication

This article was republished on August 1, 2016, to correct errors that were introduced during the typesetting process: in several places throughout the article the α was omitted from α-MSH. The publisher apologizes for the error. Please download this article again to view the correct version. The originally published, uncorrected article and the republished, corrected article are provided here for reference.

## Supporting Information

S1 File Originally published, uncorrected article. (PDF)

S2 File Republished corrected article. (PDF)
