# Supplementary figures and images for: α-MSH Stimulates Glucose Uptake in Mouse Muscle and Phosphorylates Rab-GTPase-Activating Protein TBC1D1 Independently of AMPK
Source: PLoS One. 2016 Jul 28;11(7):e0157027. doi: 10.1371/journal.pone.0157027 (PMC4965092; doi:10.1371/journal.pone.0157027)

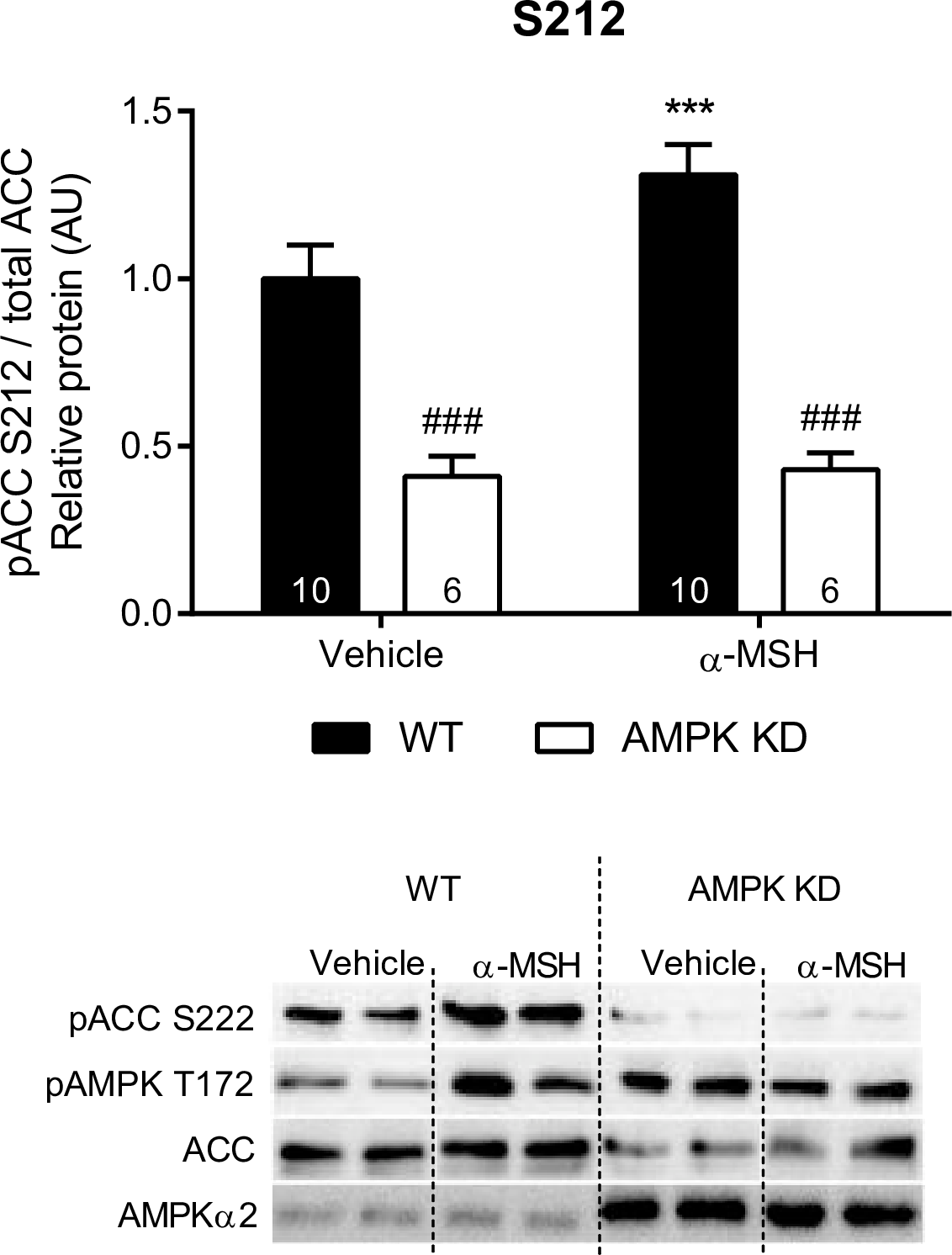

Supplement: S1 Fig — Phosphorylation of ACC S212 was measured in soleus muscle using western blotting as described. ACC phosphorylation was normalized to total ACC. We found that α-MSH stimulation phosphorylated AMPK in WT animals as previously reported by An et al. [23] (Fig 6). Phosphorylation of AMPK in AMPK KD mice was also identified, however no difference was seen between vehicle and α-MSH-treated explants. AMPK KD mice are not able to phosphorylate ACC, since ACC is a read-out of AMPK activity. We found no increase in ACC-phosphorylation after α-MSH-stimulation in AMPK KD mice, which validates the deficient kinase activity of the AMPK 2 mutant (Fig 6). It is shown that α-MSH significantly phosphorylates ACC in WT mice proving activity of AMPK. Findings are shown as a representative immunoblot and pooled data are quantified in bar graphs as arbitrary units. 2-way RM ANOVA was used to calculate statistical significance (*p < 0.05, **p < 0.01, ***p < 0.001 vs. vehicle). # indicates a significant effect of genotype (#p < 0.05, ##p < 0.01, ###p < 0.001). (TIF) [file pone.0157027.s001.tif]
